# *Plasmodium berghei*-infection induces volume-regulated anion channel-like activity in human hepatoma cells

**DOI:** 10.1111/j.1462-5822.2009.01342.x

**Published:** 2009-06-23

**Authors:** Miguel Prudêncio, Elvira T Derbyshire, Catarina A Marques, Sanjeev Krishna, Maria M Mota, Henry M Staines

**Affiliations:** 1 Unidade de Malária, Instituto de Medicina Molecular, Universidade de LisboaLisboa, Portugal; 2 Centre for Infection, Division of Cellular and Molecular Medicine, St. George's, University of LondonCranmer Terrace, London SW17 0RE, UK

## Abstract

Parasite infection can lead to alterations in the permeability of host plasma membranes. Presented here is the first demonstration that this phenomenon occurs in *Plasmodium*-infected liver cells. Using the whole-cell patch-clamp technique, volume-regulated anion channel (VRAC) activity was characterized in Huh-7 cells (a human hepatoma cell line) before and after infection with *Plasmodium berghei*. Consistent with the presence of VRACs, hypotonic bath solution induced large ion currents in Huh-7 cells that rectified outwardly, reversed close to the equilibrium potential for Cl^-^ and were inhibited by tamoxifen, clomiphene, mefloquine and 5-nitro-2, 3-(phenylpropylamino)-benzoic acid (NPPB), with IC_50_ values of 4 ± 1, 4 ± 2, 2 ± 1 and 52 ± 12 μM respectively. In isotonic conditions, initial current recordings measured in uninfected and immature (24 h post invasion) parasite-infected Huh-7 cells were similar (with conductances of 14 ± 3 versus 19 ± 5 pS/pF). However, in mature (48–72 h post invasion) parasite-infected Huh-7 cells there was a sevenfold increase in currents (with a conductance of 98 ± 16 pS/pF). The elevated currents observed in the latter are consistent with VRAC-like activity and the possible reasons for their activation are discussed.

## Introduction

Malaria causes nearly one million human deaths worldwide, annually, and is associated with morbidity in a further 250 million individuals (refer to http://www.who.int/malaria/wmr2008). The disease is caused by single-celled parasites of the genus *Plasmodium*. There is currently a critical need for novel antimalarial interventions (Cunha-Rodrigues *et al*., 2006), if the disease is to be effectively managed in coming years. This need has been heightened by a disturbing recent report that malarial parasites in Western Cambodia are resistant to artemisinins (Noedl *et al*., 2008), the most effective antimalarial drug class currently available (Krishna *et al*., 2008).

The complex life cycles of *Plasmodium* spp., which involve both a mosquito vector and a vertebrate host, can be separated into four reproductive phases (three asexual and one sexual). Two asexual reproductive phases take place within the parasite's vertebrate host in liver and red blood cells. Multiplication within red blood cells (when the clinical symptoms of the disease manifest themselves) has been studied in great detail particularly since this stage was first cultured in 1976 (Trager and Jensen, 1976). Liver stages of parasite maturation are only now revealing their secrets, with the development of new tools to aid their study [such as green fluorescence protein (GFP)-expressing parasites; Franke-Fayard *et al*., 2004; Prudencio *et al*., 2008]. The liver stages of malarial parasites, although clinically silent, are an attractive target for antimalarial vaccines and prophylactic drugs (Prudencio *et al*., 2006), especially as intervening here will prevent symptomatic malaria.

Malarial parasites alter the permeability of their host red blood cells as they develop intracellularly (Kirk, 2001). These alterations allow the transport of structurally unrelated molecules and can contribute to parasite survival by modulating nutrient uptake, waste removal and volume and ion regulation. For these reasons, some of the transport pathways that underlie these processes have been proposed as antimalarial targets (Ginsburg, 1994; Staines *et al*., 2005). Most recently, it has been shown that the electrophysiological technique of patch-clamp can be used to measure altered transport activity in infected red blood cells (Staines *et al*., 2004a; Staines *et al*., 2007). Other studies have shown that parasites can make host liver cells more resistant to pro-apoptotic stimuli (Leiriao *et al*., 2005; van de Sand *et al*., 2005). As changes in the function of transporters are important in early apoptotic pathways in liver cells (Nietsch *et al*., 2000), these pathways could be mechanistically involved in the anti-apoptotic properties of infection.

No functional transport studies have been performed in *Plasmodium*-infected liver cells. The aim of this study was to investigate the transport characteristics of parasitized liver cells and identify any differences compared with uninfected liver cells. Whole-cell currents were measured in *P. berghei*-infected Huh-7 cells (a rodent malarial parasite developing in a human hepatoma cell line), using the patch-clamp technique, and show for the first time that parasites alter the permeability of their host liver cells.

## Results

### Whole-cell currents in uninfected Huh-7 cells

The whole-cell patch-clamp technique was used to measure ion transport across the plasma membrane of individual Huh-7 cells. In preliminary experiments, using pseudo-physiological (extracellular) bath and (intracellular) pipette solutions (i.e. isotonic, high NaCl bath and high KCl pipette solutions), the predominant currents observed had the characteristics of those generated by volume-regulated anion channels (VRACs; data not shown). Currents generated by VRACs can be activated by hypotonic bath solutions and typically demonstrate moderate outward rectification (i.e. currents that are larger at positive membrane potentials than at negative membrane potentials even though equal but opposite forces are applied) and slow time-dependent current inactivation at high positive membrane potentials (> +60 mV), as described below and reviewed previously (Strange *et al*., 1996; Kirk, 1997; Nilius *et al*., 1997; Stutzin and Hoffmann, 2006). Subsequent experimental conditions were chosen to reduce the effects of other channel types on the measured currents. First, no K^+^ was present in either bath or pipette solutions to eliminate currents generated by K^+^ channels. Second, *N*-methyl-d-glucamine (NMDG^+^; a large, effectively impermeant cation) was made the predominant cation in the pipette solution to reduce cation currents moving out of the cell generated by other cation channel types. Third, the internal Ca^2+^ concentration was buffered to 100 nM to suppress currents generated by Ca^2+^-activated Cl^-^ channels, which are present in liver cells (Li and Weinman, 2002). In addition, asymmetrical Cl^-^ bath and pipette solutions were used to aid the identification of currents generated by anions. For these solutions (see *Experimental procedures*), the Cl^-^ equilibrium potential, *E*_Cl_ (i.e. the membrane potential at which the net movement of Cl^-^ across the plasma membrane is zero) was calculated to be −23 mV. Therefore, channel activity with zero current measurements close to this membrane potential is most likely anionic in nature.

**Fig. 1:**
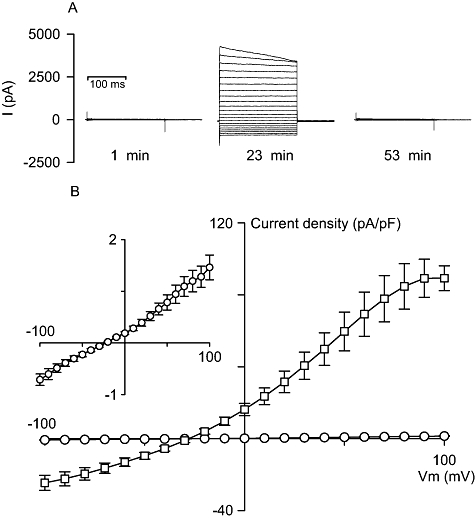
Whole-cell current recordings in uninfected Huh-7 cells under isotonic conditions. A. Representative set of current recordings in isotonic conditions, holding potential = −30 mV, which demonstrates the development and subsequent decline of currents in a proportion of uninfected Huh-7 cells. Note: times shown in the figure represent the times after whole-cell rupture. B. Current density/voltage curves for averaged late current data (measured between 140 and 200 ms) from recordings taken directly after attaining the whole-cell configuration (open circles; *n* = 30) and from recordings taken at peak current levels in a proportion of Huh-7 cells in which current levels increased spontaneously during experimentation (open squares; *n* = 5). Inset, expanded view of data generated in Huh-7 cells directly after attaining the whole-cell configuration. Late current data are shown as the mean ± SEM.

In the isotonic conditions used here, current recordings in uninfected Huh-7 cells (Fig. 1), taken within 1 min of attaining the whole-cell configuration, were small and had approximately linear or very slightly outwardly rectifying current-voltage (I–V) relationships. The average slope conductance (the change in current divided by the change in voltage), measured between +30 and +60 mV and corrected for capacitance (the latter being used to correct for differences in cell size), was 14 ± 3 pS/pF and the average reversal potential (that measured at zero current) was −27 ± 2 mV (mean ± SEM; *n* = 30). However, as recording continued, there developed in a proportion of cells a seemingly spontaneous, substantial and transient increase in the currents measured (Fig. 1). These currents peaked at 15 ± 2 min (mean ± SEM; *n* = 5) after attaining the whole-cell configuration before reducing back towards their original starting levels. They were also variable in size at their peak, often demonstrated slow time-dependent current inactivation at high positive potentials and produced outwardly rectifying I–V relationships. At their peak, the average slope conductance and reversal potential were 950 ± 170 pS/pF and −28 ± 2 mV respectively (mean ± SEM; *n* = 5). In a set of 12 consecutive experiments, in which prolonged recording was possible, this phenomenon was observed five times. These data are consistent with the transient activation in a proportion of uninfected Huh-7 cells of VRAC-like activity in isotonic conditions, a phenomenon that has been reported previously (see *Discussion*).

**Fig. 2:**
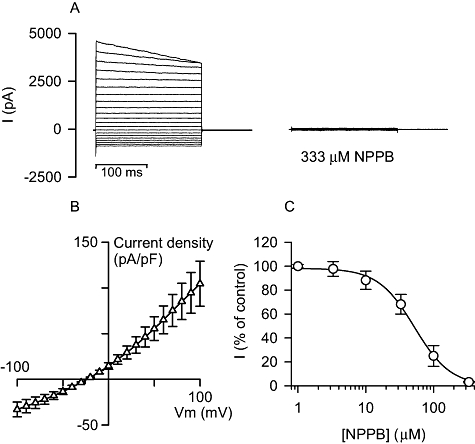
Whole-cell current recordings in uninfected Huh-7 cells under hypotonic conditions. A. Representative set of current recordings in hypotonic conditions, holding potential = −30 mV, showing the peak currents obtained in a Huh-7 cell and the subsequent effect of the addition to the bath solution of 333 μM NPPB. B. Current density/voltage curve for averaged late current data (measured between 140 and 200 ms) from recordings taken at peak current levels in Huh-7 cells exposed to hypotonic bath solution (open triangles; *n* = 7). Late current data are shown as the mean ± SEM. C. Dose–response curve for the effect of NPPB on the hypotonically activated currents in Huh-7 cells, measure at a membrane potential of +20 mV (open circles; *n* = 3). Inhibition data are shown as the mean ± SEM.

For experiments in uninfected cells, in which the spontaneous development of VRAC-like activity was not observed over a minimum 7 min period or, if observed, had reduced down to initial levels, the isotonic bath solution [320 mosmol (kg H_2_O)^−1^] was replaced with a hypotonic solution [240 mosmol (kg H_2_O)^−1^], resulting in a 25% reduction in the bath solution osmolarity. This action resulted in the generation of large currents that were essentially identical to those that occasionally developed under isotonic conditions. These currents (Fig. 2A and B) peaked at 18 ± 4 min (mean ± SEM; *n* = 7) after altering the bath solution osmolarity, were variable in size at their peak level, often demonstrated slow time-dependent current inactivation at high positive potentials and produced outwardly rectifying I–V relationships. At their peak, the average slope conductance and reversal potential were 920 ± 210 pS/pF and −28 ± 1 mV, respectively (mean ± SEM; *n* = 7). On average the currents were 70-fold larger in the positive direction and 50-fold larger in the negative direction in relation to the reversal potential than currents measured in uninfected cells in isotonic conditions. However, unlike the increased currents observed under isotonic conditions, these currents were not transient in nature and remained sufficiently stable (due to the constant osmotic gradient across the cell membrane) at their peak levels to allow pharmacological experiments to be performed.

A number of inhibitors that reduce hypotonically activated anion currents (Nilius *et al*., 1999) were tested on currents generated by hypotonic bath solution in uninfected Huh-7 cells [see Fig. 2A and C for the example of the non-specific anion transport inhibitor 5-nitro-2, 3-(phenylpropylamino)-benzoic acid (NPPB)]. The selective estrogen receptor modulators, tamoxifen and clomiphene, the blood-stage antimalarial mefloquine, and NPPB all inhibited the hypotonically activated currents in uninfected Huh-7 cells, with IC_50_ values of 4 ± 1, 4 ± 2, 2 ± 1 and 52 ± 12 μM respectively (measured at a membrane potential of +20 mV; mean ± SEM, *n* = 3). Furthermore, in anticipation of testing these inhibitors in development assays (see below) and because anion channel inhibitors are known to bind to the serum present in these types of assay (Staines *et al*., 2004b), the effect of each inhibitor was also tested in the presence of 10% v/v fetal calf serum (FCS). With serum, the IC_50_ values for tamoxifen, clomiphene, mefloquine and NPPB were 17 ± 6, 8 ± 2, 4 ± 1 and 3800 ± 2900 μM respectively (measured at a membrane potential of + 20 mV; mean ± SEM, *n* = 3).

Taken together, these data are consistent with the presence in Huh-7 cells of VRACs (see *Discussion*) and provide the basis to determine whether VRAC activity changes during Huh-7 cell infection with *Plasmodium* in isotonic conditions. In particular, it is important to note that VRAC-like activity can develop in Huh-7 cells in isotonic conditions in the absence of infection. However, the fact that this VRAC-like activity develops after attaining the whole-cell configuration (and is therefore not observed in initial current traces), has similar development characteristics to hypotonically induced VRAC activity, is transient in nature and only occurs in a proportion of cells suggests that it may be induced as a direct result of attaining the whole-cell configuration (a varying process involving application of pressure and voltage spikes until membrane rupture occurs and subsequent dilution of the cytosol by the pipette solution). Therefore, the data presented hereafter pertain predominantly to initial current recordings, before this additional VRAC-like activity develops. Nevertheless, the phenomenon has been described further, although not in detail, because it affected subsequent experimentation.

### Whole-cell currents in *P. berghei*-infected Huh-7 cells

**Fig. 3:**
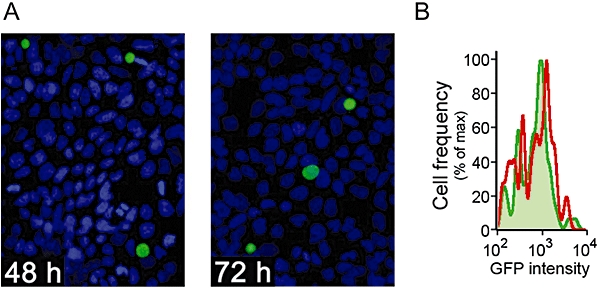
The development of live-stage *P. berghei* parasites in Huh-7 cells. A. Representative immunofluorescence microscopy images of parasitized cells at 48 h (left panel) and 72 h (right panel) post invasion. *Plasmodium* liver stages are stained in green and nuclei in blue. B. Development profile of GFP-expressing *P. berghei* parasites by flow cytometry analysis of infected cells 48 h (green line and shading) and 72 h (red line) post invasion.

Intracellular liver-stage *P. berghei* parasites develop for approximately 3 days in Huh-7 cells, forming schizonts over this time. At 24 h post invasion the parasites take up less than 5% of their host liver cell's volume but by 48 h they occupy an estimated 20–40% of liver cell volume. By 72 h schizonts are visible in culture but these mature forms do not survive trypsin digestion and were not studied further. However, at 72 h some slower developing parasites were morphologically indistinguishable from those observed at 48 h (Fig. 3) and so were treated as an identical group.

**Fig. 4:**
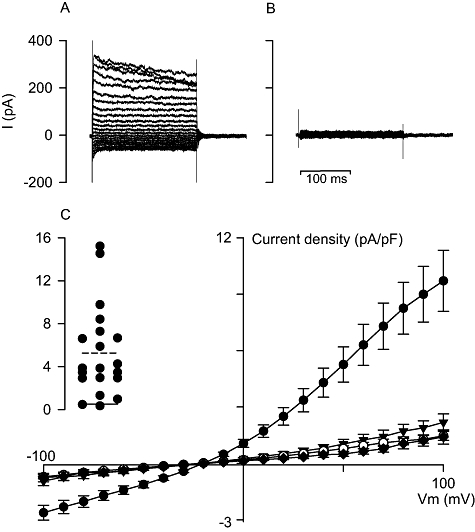
Whole-cell current recordings in *P. berghei*-infected Huh-7 cells under isotonic conditions. A. Representative current recording in a parasitized Huh-7 cell 48–72 h post invasion, holding potential = −30 mV, taken directly after attaining the whole-cell configuration. B. Representative current recording in a parasitized Huh-7 cell 48–72 h post invasion, holding potential = −30 mV, taken directly after attaining the whole-cell configuration following a 5 min pre-incubation with 333 μM NPPB. C. Current density/voltage curves for averaged late current data (measured between 140 and 200 ms) from recordings taken directly after attaining the whole-cell configuration in uninfected Huh-7 cells (open circles; *n* = 30), parasitized Huh-7 cells 24 h post invasion (closed inverted triangles; *n* = 7), parasitized Huh-7 cells 48–72 h post invasion (closed circles; *n* = 20) and parasitized Huh-7 cells 48–72 h post invasion pre-incubated (5 min) with 333 μM NPPB (closed diamonds; *n* = 4). Late current data are shown as the mean ± SEM. Inset. Individual currents measured at +50 mV for the 20 parasitized Huh-7 cells 48–72 h post invasion (solid line, mean current in uninfected Huh-7 cells; dashed line, mean current in parasitized Huh-7 cells 48–72 h post invasion).

Under isotonic conditions, current recordings taken directly after attaining the whole-cell configuration in infected Huh-7 cells that contained parasites 24 h after invasion were similar to those measured in uninfected cells (see Fig. 4C for averaged I–V data). Their average slope conductance (measured between +30 and +60 mV and corrected for capacitance) and reversal potential were 19 ± 5 pS/pF and −22 ± 2 mV respectively (mean ± SEM; *n* = 7), neither of which was significantly different from those measured in uninfected Huh-7 cells in isotonic conditions (*P*= 0.45 and 0.25 respectively; two-tailed, unpaired Student's *t*-test). From eight experiments that generated useful data in 24 h post invasion parasitized Huh-7 cells, only four whole-cell patches lasted for more than a few minutes. In 3 of these 4 experiments, increased currents were observed after continued recording in a similar fashion to those observed in a proportion of uninfected Huh-7 cells under isotonic conditions (data not shown).

Initial whole-cell currents recorded in infected Huh-7 cells containing parasites 48–72 h after invasion were larger when compared with those of both 24 h post invasion parasitized and uninfected cells, and showed slow time-dependent current inactivation at high positive potentials (Fig. 4A and C). On average, currents were sixfold larger when positive and fourfold larger when negative, with respect to the reversal potential, than currents measured in uninfected cells under isotonic conditions. Currents in individual experiments ranged from being similar to those measured in uninfected cells to 3 times the mean data for cells with parasites 48–72 h post invasion (Fig. 4C, inset). The average slope conductance (measured between +30 and +60 mV and corrected for capacitance) and reversal potential were 98 ± 16 pS/pF and −19 ± 1 mV respectively (mean ± SEM; *n* = 20), both of which were significantly different from those measured in uninfected Huh-7 cells in isotonic conditions (*P* = 2 × 10^−7^ and 0.002, respectively; two-tailed, unpaired Student's *t*-test). From 20 experiments that generated useful data in 48–72 h post invasion parasitized Huh-7 cells, 18 whole-cell patches lasted for more than a few minutes. In 12 of these 18 experiments, increased currents were observed after continued recording in a similar fashion to those observed in uninfected Huh-7 cells under isotonic conditions (data not shown). Of the six remaining, currents reduced to levels measured in uninfected cells in all but one, which remained stable (data not shown).

As the initial whole-cell currents recorded in 48–72 h post invasion parasitized Huh-7 cells were not stable, it was not possible to study their pharmacology directly. Instead, inhibitor pre-incubations (i.e. added before attaining the whole-cell configuration) were attempted to derive a pharmacological profile for these currents. Unfortunately, Huh-7 cells became friable in the presence of high concentrations of tamoxifen, clomiphene and mefloquine and lysed during whole-cell rupture. The exception was NPPB and 48–72 h post invasion parasitized cells pre-incubated with NPPB, albeit at 333 μM, for 5 min prior to patch-clamp experiments had initial whole-cell currents that were not significantly (*P*= 0.62, comparing capacitance-corrected slope conductances; two-tailed, unpaired Student's *t*-test) different from those measured in uninfected cells (Fig. 4B and C).

### Inhibitor studies on *P. berghei* development in Huh-7 cells

Having presented evidence that *Plasmodium* parasites alter the transport activity of their host liver cells, we next sought to test the effect of inhibitors used here (tamoxifen, clomiphene, mefloquine and NPPB) to interfere with parasite invasion and/or subsequent intracellular development, using a fluorescence-based, liver-stage *in vitro* assay (Prudencio *et al*., 2008). In all cases, the presence of these compounds did not alter the number of GFP positive Huh-7 cells (i.e. the number of infected liver cells) when compared with control values (*P* > 0.05; anova with Tukey's post test), consistent with the process of cell invasion by parasites (as opposed to their subsequent intracellular development) being unaffected by these compounds (data not shown).

**Fig. 5:**
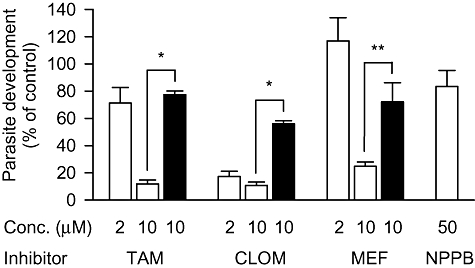
Effect of current inhibitors on the development of *P. berghei* parasites in Huh-7 cells. Effect of current inhibitors added to Huh-7 cells 1 h before invasion (open bars) and 24 h post invasion (closed bars) on parasite development measured at 48 h post invasion. Mean (geometric) GFP intensity was used to estimate parasite development and is presented as a percentage of the GFP intensity measured in paired control experiments performed in the absence of any inhibitors. To calculate development data measured over the 24–48 h period, the GFP intensity of parasitized Huh-7 cells measured at 24 h post invasion (approximately 15% of that measured at 48 h post invasion in control experiments) was first subtracted from each data set, including the control data. Uncorrected geometrical mean (and range) GFP-intensities for infected cells (*n* = 3) measured at 2 (background), 24 and 48 h post invasion were 26 (25–27), 79 (46–160) and 340 (159–651) respectively. TAM, tamoxifen; CLOM, clomiphene; MEF, mefloquine; NPPB, 5-nitro-2, 3-(phenylpropylamino)-benzoic acid. *P < 0.005 and **P < 0.05 (two-tailed, unpaired Student's *t*-test on log-transformed data).

Tamoxifen, clomiphene and mefloquine (all 10 μM) inhibited the intracellular development of liver-stage parasites by more than 75% over the first 48 h post invasion, when added to the cells 1 h before the sporozoites (Fig. 5, open bars). Clomiphene also inhibited development by more than 80% at a lower concentration (2 μM). NPPB at a concentration of 50 μM (the highest concentration tested that was not detrimental to uninfected Huh-7 cells) had little effect on the intracellular development of liver-stage parasites, which, in part, is consistent with its reduced effectiveness in the presence of serum (as reported above). However when 10 μM concentrations of tamoxifen, clomiphene and mefloquine were added to parasitized cells 24 h post invasion, their effect on parasite development over the subsequent 24 h (when elevated currents are observed) was significantly lower than the overall effect of adding them shortly before infection (Fig. 5, closed bars). It should be noted that 10 μM concentrations of tamoxifen, clomiphene and mefloquine inhibited VRAC activity in the presence of serum (as used in the development assays) by 25%, 60% and 80% respectively. Higher concentrations of each inhibitor (33 μM) that would result in maximal inhibition of VRAC activity in the presence of serum were also tested on parasite development but were toxic to Huh-7 cells (data not shown).

## Discussion

The aim of this study was to establish if intracellular liver-stage malarial parasites alter the permeability of their host cell's plasma membrane, using the whole-cell patch-clamp technique. In particular, we have focused on currents generated by VRACs. It was therefore appropriate to characterize, for the first time, VRAC activity in uninfected Huh-7 cells, using the whole-cell patch-clamp technique.

### VRAC activity in Huh-7 cells

Volume-regulated anion channels are found in nearly all cell types (Nilius *et al*., 1997), including hepatocytes and hepatoma cells (Li and Weinman, 2002). In addition to volume regulation (specifically volume regulatory decrease), they are thought to be involved in several cellular processes including proliferation and apoptosis (Stutzin and Hoffmann, 2006). Although their molecular identity has remained enigmatic, the functional characteristics of VRACs are well defined. Typically, currents generated by VRACs demonstrate moderate outward rectification and slow time-dependent inactivation at high positive potentials with a selectivity profile of I^-^ > Br^-^ > Cl^-^ (corresponding to Eisenman's sequence I). VRACs are blocked by a range of, generally non-specific, channel inhibitors including the four used in this study (Nilius *et al*., 1999). VRACs are also proposed to transport, albeit at lower rates, a range of structurally unrelated, charged and uncharged, small solutes, including the organic osmolytes (also involved in volume regulatory decrease) taurine and sorbitol (Strange *et al.*, 1996; Kirk, 1997).

Shortly after osmotic reduction of the bath solution, Huh-7 cells developed, over several minutes, large outwardly rectifying currents that reversed close to the equilibrium potential for Cl^-^, often demonstrated some degree of time-dependent inactivation above +60 mV and were blocked by known inhibitors of VRACs including NPPB and tamoxifen. These data are consistent with similar observations made in previous whole-cell patch-clamp studies in a range of hepatocyte and hepatoma cells (Jackson *et al*., 1996; Meng and Weinman, 1996; Bodily *et al*., 1997; Feranchak *et al*., 2000; Wondergem *et al*., 2001) and in other cell types (Nilius *et al*., 1997) and, suggest VRACs underlie the hypotonically activated currents in Huh-7 cells.

Transient VRAC-like activity was also observed to develop in a proportion (> 40%) of whole-cell experiments in uninfected Huh-7 cells in isotonic conditions after attaining the whole-cell configuration. A similar phenomenon, albeit occurring in nearly all whole-cell experiments and over a much faster time-scale, has been reported previously in T lymphocytes (Schumacher *et al*., 1995) and spontaneous development of VRAC-like activity in isotonic conditions has also been observed in a proportion of whole-cell recordings in AML12 cells (a mouse liver cell line; Wondergem *et al*., 2001) and rat hepatocytes (Meng and Weinman, 1996). As noted in the *Results* section, the triggering of this type of event can be directly linked to the process of attaining the whole-cell configuration and thus is unlikely to be of any physiological consequence. While the data possibly suggest that infected liver cells might be more susceptible to this phenomenon (occurring in approximately 70% of parasitized Huh-7 cells), it was only described further to show how it affected the study of currents measured in initial traces, which are more likely to be physiologically relevant.

### Altered VRAC-like activity in *P. berghei*-infected Huh-7 cells

In isotonic conditions, currents measured in initial whole-cell recordings in *P. berghei*-infected Huh-7 cells 24 h post invasion were low and not different from those measured in uninfected cells. However, in parasitized cells, from 48 h post invasion, averaged currents were elevated significantly, demonstrating that parasites at a late stage of hepatic development modify their host cell's permeability. The variable magnitude of these currents shows that altered permeability is not a uniform response to infection, with 20% of parasitized cells having currents no larger than uninfected Huh-7 cells. The record characteristics of these parasite-induced currents and their (limited) pharmacological profiling are consistent with those produced by VRACs. The only difference, other than the fact that these currents were observed in initial current recordings in isotonic conditions, was a significant shift of the reversal potential (from −28 to −19 mV), although this is still consistent with an anion conductance as the *E*_Cl_ is calculated at −23 mV for the conditions used here. The reason for this shift is not clear but may result from the incomplete dilution of the cytosol by the pipette solution at the time of the initial recording (in continuous recordings the reversal potential tended to move back towards −28 mV but, as reported above, currents also tended to alter at the same time). The magnitude of the currents suggests that their activation is controlled as even the highest modified currents measured were smaller than the average observed in uninfected cells submitted to a 25% reduction in osmolarity and on average only 10% of the maximal currents reported in this study were recorded in initial traces in mature infected liver cells.

The modified currents in parasitized Huh-7 cells differ from those observed in *P. falciparum* and *P. berghei*-infected erythrocytes (Decherf *et al*., 2004; Huber *et al*., 2004; Staines *et al*., 2007). Under similar conditions to those used here, currents in *P. falciparum*-infected erythrocytes are predominantly inwardly rectifying (i.e. greater currents at negative potentials than positive potentials) and stable over time, although time-dependent inactivation can develop at negative potentials (Staines *et al*., 2003). It is possible that additional anion channels could underlie the current measured at negative potentials in parasitized Huh-7 cells with increased anion conductance. However, given that this component (i.e. the currents at negative potentials) is relatively small, it would not account for much of the elevated currents, although it could be important at physiological, resting membrane potentials (−30 to −40 mV). Under certain conditions (e.g. in the presence of serum), outwardly rectifying currents have been measured in both *P. falciparum* and *P. berghei*-infected erythocytes (Huber *et al*., 2004; Staines *et al*., 2006) but these again have different characteristics to those measured here, having time-dependent current inactivation at negative potentials and none at high positive potentials. In addition, chicken erythrocytes infected with *P. gallinaceum*, although having altered electroneutral and electrogenic solute transport, do not activate VRACs (Thomas *et al*., 2001; Staines *et al*., 2002), even though cell swelling does induce them (note that, unlike avian erythrocytes, mammalian erythrocytes are some of the few cell types that do not activate VRACs upon cell swelling).

### Is altered VRAC activity required for parasite survival or a host response to infection?

In *Plasmodium*-infected erythrocytes, altered host cell membrane permeability is thought to be beneficial to the survival of the parasite. However, while 10 μM tamoxifen, clomiphene and mefloquine inhibited parasite development, the fact that this occurred to a greater extent over the first 24 h than second 24 h of infection (the latter being when elevated VRAC activity is observed in parasitized cells) suggests that VRAC may not be important to parasite survival. This can be added to the fact that mefloquine, a highly effective blood-stage antimalarial, is not considered an effective liver-stage antimalarial *in vivo* (Boulard *et al.*, 1986). Nevertheless, it should be noted that it was not possible to study parasite development at inhibitor concentrations that would block VRAC activity maximally and, given that parasites only activate approximately 10% on average of total reported VRAC activity, the possibility still exists that parasite development benefits from VRAC activation. It was also not possible to determine the subsequent ability of parasites that developed in the presence of VRAC inhibitors to invade and grow in erythrocytes (in case delayed death effects occurred).

The ability of the selective estrogen receptor modulators, tamoxifen and in particular clomiphene, to inhibit liver-stage parasite development might warrant further investigation. Clomiphene has previously been reported to inhibit blood-stage parasite growth *in vitro* by 80% at 10 μM (Staines *et al*., 2004c) and here inhibited liver-stage development by 80% at 2 μM. The latter is not much higher than clomiphene's therapeutic plasma concentration range of 0.1–0.4 μM (Young *et al.*, 1999). The data presented here do little to suggest a possible mechanism of action.

Volume-regulated anion channel activity in the absence of cell swelling is an early step in apoptosis (Stutzin and Hoffmann, 2006), a process that leads to a protective immune response after hepatocyte infection by irradiated malarial sporozoites (van Dijk *et al.*, 2005). Therefore, the parasite-induced VRAC-like activity reported here could be a host response to parasite infection. However, two reports (Leiriao *et al.*, 2005; van de Sand *et al.*, 2005) have also presented evidence that *P. berghei*-infected HepG2 cells (another human hepatoma cell line) and infected mice hepatocytes *in vivo* are highly resistant to apoptosis, as assessed by a lack of caspase activation and lamin and DNA degradation, induced by peroxide and tumour necrosis factor treatment [which, incidentally, also induce VRAC activity in HeLa and HTC rat hepatoma cells (Shimizu et al., 2004; Varela *et al.*, 2007)]. So how might these data fit with those reported here? The growing parasite may cause the host to attempt to induce apoptosis that results in elevated VRAC activity, with the parasite blocking an apoptosis step between VRAC activation and caspase activation. Further investigations aimed at interventional therapies to reduce the anti-apoptotic properties of infection (resulting in an improved immune response to liver-stage malarial parasites) may benefit from detailed transport studies to define mechanisms.

### Conclusions

Here we have demonstrated for the first time that intracellular liver-stage plasmodial parasites alter the permeability of their host's plasma membrane. The data are consistent with mature parasitized cells having elevated anion currents produced by VRAC-like activity in isotonic conditions (and are different from those reported in *Plasmodium*-infected erythrocytes). At present, the reasons behind this phenomenon are not clear but it may be required by the parasite for survival or could result from the host's response to fight infection (attempting to induce apoptosis).

## Experimental procedures

### Materials

Tamoxifen, clomiphene, mefloquine and NPPB were obtained from Sigma (Dorset, UK). Culture medium and supplements were obtained from Invitrogen (Renfrewshire, UK). Stock inhibitor solutions were made in dimethyl sulfoxide (DMSO).

### Cell culture

Huh-7 cells, a human hepatoma cell line, were cultured in 1640 RPMI medium supplemented with 10% v/v FCS, 1% v/v nonessential amino acids, 1% v/v penicillin/streptomycin, 2 mM glutamine and 10 mM 4-(2-hydroxyethyl)-1-piperazineethanesulfonic acid (Hepes), pH 7, and maintained at 37°C with 5% CO_2_.

To obtain parasitized cells for electrophysiological measurements, Huh-7 cells (5 × 10^4^ per well) were seeded in 24-well plates the day before infection. Cells were infected by addition of specific numbers of sporozoites (typically 30 000), followed by centrifugation at 1700 *g* for 7 min at 37°C. GFP-expressing *P. berghei* (parasite line 259 cL2) sporozoites were obtained by disruption of the salivary glands of freshly dissected infected female *Anopheles stephensi* mosquitoes.

### Electrophysiological recording

Patch pipettes (tip resistances 3–8 MΩ) were prepared from borosilicate glass capillaries pulled and polished on a Werner Zeitz DMZ programmable puller (Augsburg, Germany). The isotonic bath solution contained (in mM) NaCl 105, CaCl_2_ 1.5, MgCl_2_ 1, d-mannitol 90, d-glucose 10, Hepes 10, adjusted to pH 7.4, 320 ± 5 mosmol (kg H_2_O)^−1^, while the d-mannitol was omitted to produce the hypotonic bath solution, 240 ± 5 mosmol (kg H_2_O)^−1^. The pipette solution contained (in mM) NMDG-Cl 40, NMDG-aspartate 100, MgCl_2_ 1, CaCl_2_ 1.93, ethylene glycol-*O*,*O*′-bis(2-aminoethyl)-*N*,*N*,*N*′,*N*′-tetraacetic acid (EGTA) 4, Na_2_ATP 4, Hepes 10, adjusted to pH 7.2, 290 ± 5 mosmol (kg H_2_O)^−1^.

Prior to experimentation cells were separated by exposure to 0.05% w/v trypsin/0.02% w/v EDTA in FCS-free culture medium for 5 min at 37°C and 2 ml aliquots of washed cell suspension were placed into 3 cm^2^ Petri dishes and left, while the cells settled. Cells were then washed 3 times in bath solution. Single parasitized Huh-7 cells for patch-clamp experiments were identified by epi-fluorescence microscopy, using an appropriate filter set for GFP.

The ruptured patch, whole-cell, voltage-clamp configuration was used to record membrane currents (as detailed previously, see for example Staines *et al*., 2003). All experiments were performed at room temperature (∼23°C). An Ag-AgCl pellet was used as the reference electrode. The liquid junction potential between the bath and pipette solutions was calculated and compensated for. Membrane seals (5–50 GΩ) were obtained by the application of suction to the pipette (1–2 kPa) followed by the imposition of a negative pipette potential (−5 to −30 mV). Cell rupture was attained by a short burst of strong suction and/or a short high voltage pulse and the configuration assessed by a decrease in access resistance and the development of a capacitance transient. Both were recorded for each experiment using the ‘Membrane Test’ set-up in pCLAMP. The capacitances for uninfected (*n* = 32) and parasitized (*n* = 33) Huh-7 cells were 28 ± 1 and 34 ± 2 pF respectively (mean ± SEM). Whole-cell currents were recorded using an Axopatch 200B amplifier (digitized at 10 kHz and filtered at 5 kHz with a 4-pole Bessel filter), with voltage command protocols generated and the currents analysed using the pCLAMP software suite (Version 10, Axon Instruments, USA). Whole-cell I–V curves were obtained by evoking a series of test potentials from −100 to +100 mV in 10 mV steps for 200 ms from a holding potential of −30 mV. Data for the construction of I–V curves were measured over the last 60 ms of the current records (i.e. 140–200 ms).

### Parasite development assays

Intracellular parasite development was determined by measuring GFP intensity in cells infected with GFP-expressing *P. berghei* parasites by fluorescence-activated cell sorting (FACS), as described previously (Prudencio *et al*., 2008). Briefly, cell samples for FACS analysis were washed with 1 ml of phosphate-buffered saline (PBS), incubated with 100 μl of trypsin for 5 min at 37°C and collected in 400 μl of 10% v/v FCS in PBS at the selected time points post sporozoite addition. Cells were then centrifuged at 0.1 *g* for 5 min at 4°C and re-suspended in 150 μl of 2% v/v FCS in PBS. Cells were analysed on a Becton Dickinson FACScalibur with the appropriate settings for the fluorophore used. Data acquisition and analysis were carried out using the CELLQuest (version 3.2.1fl1, Becton Dickinson) and FlowJo (version 6.3.4, FlowJo) software packages respectively.

### Immunofluorescence

Huh-7 cells were fixed with 4% paraformaldehyde in PBS for 20 min and incubated in blocking buffer (3% w/v bovine serum albumin, 10% v/v FCS, 100 mM glycine, 0.1% w/v saponin in PBS) for 1 h followed by incubation with monoclonal antibody 2E6 against *P. berghei* HSP70 diluted in the same buffer. Cells were then washed with 0.1% w/v saponin in PBS and incubated with a secondary antibody diluted in blocking buffer (Anti-Mouse Alexa488, Molecular Probes) for 30 min. Nuclei were stained with DAPI. Images were acquired with a Leica DM5000B fluorescence microscope and processed using Adobe Photoshop.
